# A Transferable IncC-IncX3 Hybrid Plasmid Cocarrying *bla*_NDM-4_, *tet*(X), and *tmexCD3-toprJ3* Confers Resistance to Carbapenem and Tigecycline

**DOI:** 10.1128/mSphere.00592-21

**Published:** 2021-08-04

**Authors:** Aki Hirabayashi, Trung Duc Dao, Taichiro Takemura, Futoshi Hasebe, Le Thi Trang, Nguyen Ha Thanh, Hoang Huy Tran, Keigo Shibayama, Ikuro Kasuga, Masato Suzuki

**Affiliations:** a Antimicrobial Resistance Research Center, National Institute of Infectious Diseases, Tokyo, Japan; b Vietnam Research Station, Center for Infectious Disease Research in Asia and Africa, Institute of Tropical Medicine, Nagasaki University, Nagasaki, Japan; c National Institute of Hygiene and Epidemiology, Hanoi, Vietnam; d Nagoya University Graduate School of Medicine, Nagoya, Japan; e Vietnam-Japan University, Vietnam National University, Hanoi, Vietnam; f Department of Urban Engineering, Graduate School of Engineering, The University of Tokyo, Tokyo, Japan; Antimicrobial Development Specialists, LLC

**Keywords:** carbapenem, tigecycline, *bla*_NDM-4_, *tex*(X), *tmexCD3-toprJ3*

## Abstract

Tigecycline is a last-resort antimicrobial against carbapenemase-producing *Enterobacterales* (CPE). However, mobile tigecycline resistance genes, *tet*(X) and *tmexCD-toprJ*, have emerged in China and have spread possibly worldwide. Tet(X) family proteins function as tigecycline-inactivating enzymes, and TMexCD-TOprJ complexes function as efflux pumps for tigecycline. Here, to the best of our knowledge we report a CPE isolate harboring both emerging tigecycline resistance factors for the first time. A carbapenem- and tigecycline-resistant Klebsiella aerogenes strain, NUITM-VK5, was isolated from an urban drainage in Vietnam in 2021, and a plasmid, pNUITM-VK5_mdr, cocarrying *tet*(X) and *tmexCD3-toprJ3* along with the carbapenemase gene *bla*_NDM-4_ was identified in NUITM-VK5. pNUITM-VK5_mdr was transferred to Escherichia coli by conjugation and simultaneously conferred high-level resistance against multiple antimicrobials, including carbapenems and tigecycline. An efflux pump inhibitor reduced TMexCD3-TOprJ3-mediated tigecycline resistance, suggesting that both tigecycline resistance factors independently and additively contribute to the high-level resistance. The plasmid had the IncX3 and IncC replicons and was estimated to be a hybrid of plasmids with different backbones. Unlike IncX3 plasmids, IncC plasmids are stably maintained in an extremely broad range of bacterial hosts in humans, animals, and the environment. Thus, the future global spread of multidrug resistance plasmids such as pNUITM-VK5_mdr poses a public health crisis.

**IMPORTANCE** Tigecycline is important as a last-resort antimicrobial and effective against antimicrobial-resistant bacteria, such as carbapenem-producing *Enterobacterales* (CPE), whose infections are difficult to treat with antimicrobials. Since 2019, mobile tigecycline resistance genes, *tet*(X) and *tmexCD-toprJ*, and their variants have been reported mainly from China, and it has become important to understand their epidemiological situation and detailed genetic mechanisms. In this study, we identified a bacterial isolate coharboring *tet*(X) and *tmexCD-toprJ* on the same plasmid. A Klebsiella aerogenes isolate in Vietnam carried both these tigecycline resistance genes on a transferable plasmid leading to high-level resistance to multiple clinically important antimicrobials, including carbapenem and tigecycline, and could actually transfer the plasmid to other bacteria. The spread of such a multidrug resistance plasmid among bacterial pathogens should be of great concern because there are few antimicrobials to combat bacteria that have acquired the plasmid.

## INTRODUCTION

Tigecycline, a semisynthetic glycylcycline, is considered a last-resort antimicrobial against infections caused by multidrug-resistant (MDR) Gram-negative bacteria, including carbapenemase-producing *Enterobacterales* (CPE) (1, 2). Carbapenemase genes, including *bla*_NDM_, *bla*_KPC_, *bla*_IMP_, *bla*_VIM_, and *bla*_OXA-48_, are often carried on plasmids, which are self-transmissible via bacterial conjugation (3). Recently, variants of a mobile tigecycline resistance gene, *tet*(X), encoding flavin-dependent monooxygenases that catalyze tigecycline degradation, emerged in *Enterobacterales* and Acinetobacter species in China and other countries (4–6). Furthermore, a mobile tigecycline resistance gene cluster, *tmexCD-toprJ*, encoding the resistance-nodulation-cell division (RND) efflux pump that excretes multiple antimicrobials, such as tetracyclines including tigecycline, cephalosporins, fluoroquinolones, and aminoglycosides, emerged predominantly in *Enterobacterales*: *tmexCD1-toprJ1* was identified in plasmids in Klebsiella species isolated from humans and livestock in China and Vietnam (7–9). *tmexCD2-toprJ2* was identified in the plasmid and chromosome of Raoultella ornithinolytica isolated from a human in China (10). *tmexCD3-toprJ3* was identified in the chromosome of Proteus mirabilis isolated from livestock feces in China (11). In this study, to the best of our knowledge we identified a CPE isolate harboring both mobile tigecycline resistance genes, *tet*(X) and *tmexCD-toprJ*, for the first time and characterized a transferable IncC-IncX3 hybrid plasmid cocarrying *bla*_NDM-4_, *tet*(X), and *tmexCD3-toprJ3* in Klebsiella aerogenes isolated in Vietnam.

## RESULTS AND DISCUSSION

A carbapenem- and tigecycline-resistant K. aerogenes isolate, NUITM-VK5, was obtained from an urban drainage in Hanoi, Vietnam, in March 2021. K. aerogenes (formerly Enterobacter aerogenes) is an important human opportunistic pathogen and a frequent cause of nosocomial infections. The result of antimicrobial susceptibility testing (AST) of K. aerogenes NUITM-VK5 showed that NUITM-VK5 was resistant to almost all antimicrobials tested (Table 1). The MICs of tigecycline, tetracyclines, carbapenems, cephalosporins, fluoroquinolone, and aminoglycosides (other than amikacin) were more than 128 mg/liter (R) and that of colistin was 4 mg/liter (R), whereas that of amikacin was 32 mg/liter (I).

**TABLE 1:** MICs of antimicrobials against K. aerogenes NUITM-VK5 and its transconjugant of E. coli J53 harboring the plasmid pNUITM-VK5_mdr (J53/pNUITM-VK5_mdr)^*a*^

Strain	MIC (mg/liter)													
	TGC (+NMP)	MIN	DOX	TET	IPM	MEM	CTX	CAZ	CIP	AMK	GEN	TOB	STR	CST
K. aerogenes NUITM-VK5	>128 (32)	>128	>128	>128	>128	>128	>128	>128	>128	32	>128	>128	>128	4
E. coli J53	0.5 (0.5)	4	4	1	0.125	0.064	0.125	0.25	0.016	0.5	0.125	0.5	1	0.25
E. coli J53/pNUITM-VK5_mdr	128 (32)	128	128	>128	16	16	>128	128	32	16	2	32	128	0.25
E. coli ATCC 25922	0.125 (0.125)	1	1	2	0.25	0.032	0.125	0.5	0.008	2	0.5	1	0.5	0.25

a The efflux pump inhibitor 1-(1-naphthylmethyl)-piperazine (NMP) was used at 75 mg/liter. Abbreviations: TGC, tigecycline; MIN, minocycline; DOX, doxycycline; TET, tetracycline; IPM, imipenem; MEM, meropenem; CTX, cefotaxime; CAZ, ceftazidime; CIP, ciprofloxacin; AMK, amikacin; GEN, gentamicin; TOB, tobramycin; STR, streptomycin; CST, colistin.

Short-read sequence analysis of K. aerogenes NUITM-VK5 with MiSeq constructed the draft genome consisting of 181 contigs (5.9 Mbp, accession no. BPFV01000000). Multilocus sequence type (MLST) analysis showed that NUITM-VK5 belonged to sequence type 4 (ST4). Detection of antimicrobial resistance (AMR) genes using ResFinder v4.1 with the modified library including nucleotide sequences of known variants of *tmexCD-toprJ* revealed that NUITM-VK5 harbored *bla*_NDM-4_, *tet*(X), and *tmexCD3-toprJ3* along with multiple clinically relevant AMR genes, such as *bla*_CTX-M-14_ (extended-spectrum β-lactamase gene), *qnrS1* (fluoroquinolone resistance gene), *aac(6′)-lb-cr* (aminoglycoside resistance gene), and *cfr* (phenicol/lincosamide resistance gene). NUITM-VK5 was colistin resistant but did not harbor known mobile colistin resistance genes, such as *mcr*. The coding sequences of *tet*(X) and *tmexCD3-toprJ3* in NUITM-VK5 were highly identical to those of *tet*(X) in Escherichia coli 47EC (accession no. MK134376) isolated from a pig in China in 2018 and *tmexCD3-toprJ3* in P. mirabilis RGF134-1 (accession no. CP066833) isolated from a pig in China in 2019, respectively (see Fig. S1 in the supplemental material). The identity for *tet*(X) was 97.7% (1,131/1,158 nucleotides [nt]), resulting in 12 amino acid substitutions (I356A, K359R, E363A, T366I, Q367I, I370T, K374S, P375L, T378S, Q381K, L383M, and V385L). For *tmexC3*, the identity was 99.7% (1,161/1,164 nt), resulting in three amino acid substitutions (Q187H, T256M, and A386T). For *tmexD3*, the identity was 99.9% (3,133/3,135 nt), resulting in two amino acid substitutions (V610L and L611F). For *toprJ3*, the identity was 100% (1,434/1,434 nt).

10.1128/mSphere.00592-21.1 FIG S1 Multiple sequence alignment analyzed by MAFFT v7.480. (A) Comparison between gene products of *tet*(X) in K. aerogenes NUITM-VK5 (accession no. BPFV01000000) and the reference gene in E. coli 47EC (accession no. MK134376). Twelve amino acid substitutions (I356A, K359R, E363A, T366I, Q367I, I370T, K374S, P375L, T378S, Q381K, L383M, and V385L) were found. (B) Comparison between gene products of *tmexC3* in K. aerogenes NUITM-VK5 and the reference sequence in P. mirabilis RGF134-1 (accession no. CP066833). Three amino acid substitutions (Q187H, T256M, and A386T) were found. (C) Comparison between gene products of *tmexD3* in K. aerogenes NUITM-VK5 and the reference sequence in P. mirabilis RGF134-1. Two amino acid substitutions (V610L and L611F) were found. Download FIG S1, PDF file, 0.03 MB. Copyright © 2021 Hirabayashi et al. 2021 Hirabayashi et al. https://creativecommons.org/licenses/by/4.0/ This content is distributed under the terms of the Creative Commons Attribution 4.0 International license.

**FIG 1:**
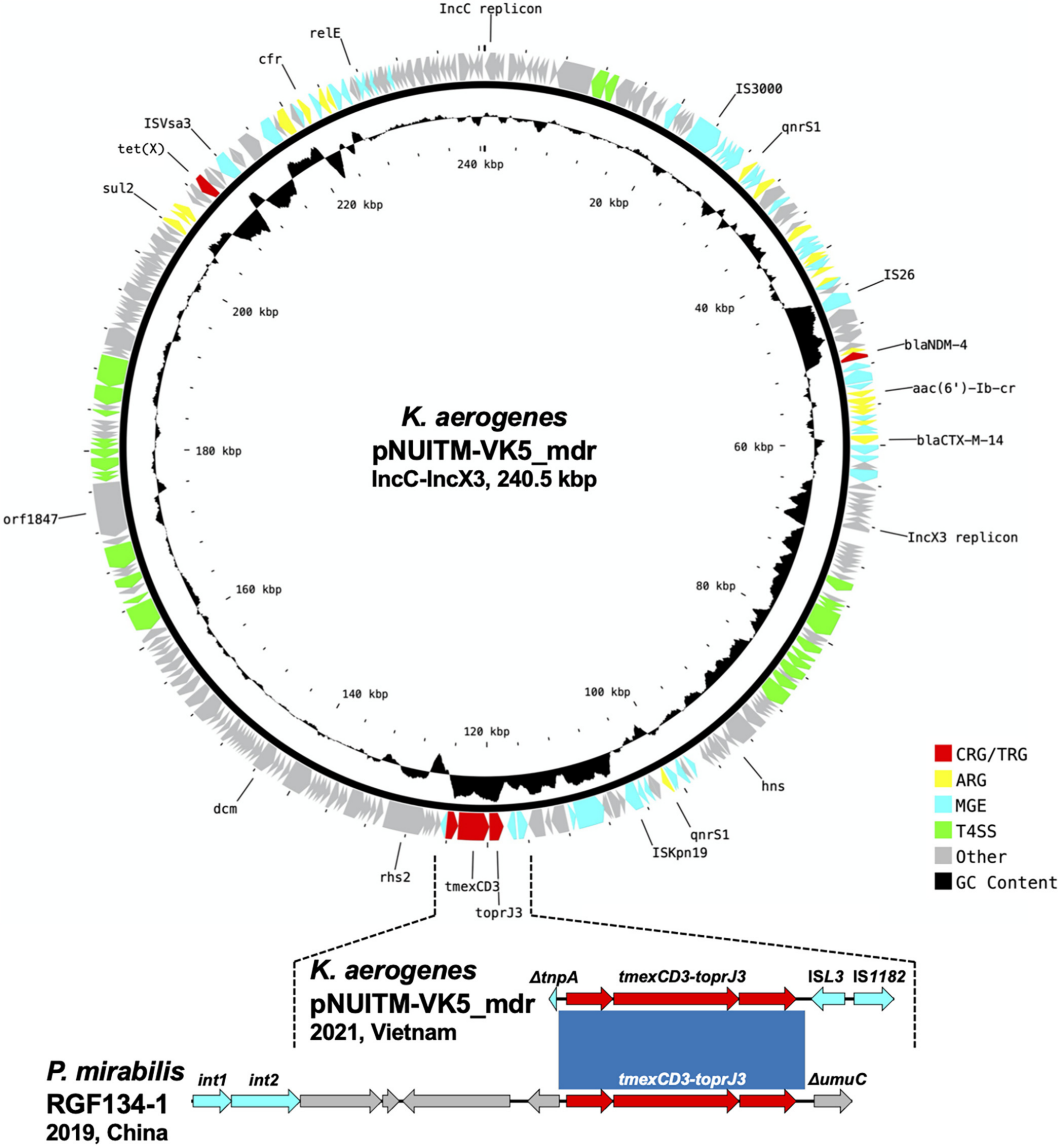
(Upper panel) Circular representation of a 240.5-kbp IncC-IncX3 hybrid plasmid, pNUITM-VK5_mdr, cocarrying multiple antimicrobial resistance genes including *bla*_NDM-4_, *tet*(X), and *tmexCD3-toprJ3* in K. aerogenes NUITM-VK5 isolated in Vietnam in 2021. (Lower panel) Linear comparison of *tmexCD3-toprJ3*-containing regions in K. aerogenes pNUITM-VK5_mdr and in a chromosome of P. mirabilis RFG134-1 isolated in China in 2019. Red, yellow, cyan, green, gray, and black indicate carbapenem and tetracycline resistance genes (CRG/TRG), other AMR genes (ARG), mobile gene elements (MGE), type IV secretion system (T4SS)-associated genes involved in conjugation, other coding sequences (Other), and GC content, respectively. The blue color in comparison of sequences indicates almost 100% identity.

The subsequent long-read sequence analysis of K. aerogenes NUITM-VK5 with MinION successfully constructed a circular plasmid, pNUITM-VK5_mdr (240.5 kbp, accession no. LC633285), cocarrying the aforementioned AMR genes detected in the draft genome (Fig. 1). Detection of plasmid replicons with PlasmidFinder v2.1 revealed that pNUITM-VK5_mdr had two replicons classified to incompatibility groups C (IncC) and X3 (IncX3). *bla*_NDM-4_, *tet*(X), and *tmexCD3-toprJ3* were carried at different locations on pNUITM-VK5_mdr, and the GC contents of the regions surrounding those AMR genes [61.6% for *bla*_NDM-4_, 37.4% for *tet*(X), and 66.0% for *tmexCD3-toprJ3*] were different from the average of the whole plasmid (51.4%), suggesting that these regions were acquired via horizontal gene transfer (HGT) mediated by mobile gene elements (MGEs) (Fig. 1) (7, 12). *tmexCD3-toprJ3* was flanked by two MGEs, IS*L3* and IS*1182*, in pNUITM-VK5_mdr; this genetic structure was different from the *tmexCD3-toprJ3*-surrounding region in the chromosomal SXT/R391 integrative conjugative element (ICE) in P. mirabilis RGF134-1 (Fig. 1, bottom) (11). *bla*_NDM-4_ and *tet*(X) in pNUITM-VK5_mdr were estimated to be acquired via HGT mediated by MGEs IS*26* and IS*Vsa3*, respectively, as previously reported (13, 14).

A bacterial conjugation assay using E. coli J53 as the recipient strain showed that K. aerogenes NUITM-VK5 transferred pNUITM-VK5_mdr to J53 at a frequency of 1.0 × 10^−6^ after overnight coculture at 37°C. The transconjugant strain (J53/pNUITM-VK5_mdr) was confirmed to coharbor *bla*_NDM-4_, *tet*(X), and *tmexCD3-toprJ3* by PCR and was resistant to almost all antimicrobials, including carbapenems and tigecycline (Table 1). The transconjugant strain was susceptible only to colistin, although parental NUITM-VK5 was resistant, suggesting that colistin resistance of NUITM-VK5 was due to factors, including chromosomal gene mutations, other than pNUITM-VK5_mdr. The addition of 1-(1-naphthylmethyl)-piperazine (NMP), which has inhibitory activity for RND efflux pumps (15), reduced the MIC of tigecycline from 128 mg/liter or higher to 32 mg/liter in NUITM-VK5 and the transconjugant strain (Table 1). Since the MIC of tigecycline against E. coli J53 was 0.5 mg/liter, 32 mg/liter for the MIC against J53/pNUITM-VK5_mdr in the presence of NMP was still high, suggesting that TMexCD3-TOprJ3 and Tet(X) contributed to tigecycline resistance independently and additively and that Tet(X) remained active even when the RND efflux pump was inhibited. On the other hand, consistent with the previous study (7), the addition of NMP did not affect the MIC of meropenem against NUITM-VK5 and J53/pNUITM-VK5_mdr, suggesting that TMexCD3-TOprJ3 does not contribute to carbapenem resistance (data not shown).

BLASTn analysis using megablast showed that no plasmid showed more than 90% identity in more than 80% of regions with the IncC-IncX3 hybrid plasmid pNUITM-VK5_mdr in the NCBI Nucleotide collection (nr/nt) database. A comparison with the known IncC plasmids showed that the IncX3 backbone of pNUITM-VK5_mdr might include the 83.5-kb region between IS*3000* and IS*Kpn19* and the IncC backbone might include the remaining region (Fig. 1). In this case, *bla*_NDM-4_ and *tet*(X) were derived from the IncX3 and IncC backbones, respectively, and *tmexCD3-toprJ3* was located at the boundary of the two backbones. IncC (formerly IncA/C_2_) is divided into type 1 and type 2 (16, 17). The IncC backbone of pNUITM-VK5_mdr would belong to type 2 as it had *rhs2* and *orf1847*, which are characteristic genetic markers of type 2 (Fig. 1).

IncC, which is involved in the spread of AMR genes, has an extremely broad host range of *Gammaproteobacteria* (18), whereas IncX3, which is also involved in the spread of AMR genes, such as *bla*_NDM_, has a narrow host range of *Enterobacterales* (19). The combination of two incompatibility groups resulted in an IncC-IncX3 hybrid plasmid, which is expected to possess an increased risk of carrying more AMR genes and spreading more stably and efficiently among various bacterial species in humans, animals, and the environment. Moreover, the acquisition of an additional RND efflux pump, TMexCD3-TOprJ3, could allow the bacterial host to survive under a variety of conditions such as antimicrobial exposure, leading to further accumulation of AMR genes into the host genome via HGT (20).

### Conclusions.

The future global spread of such a broad-host-range self-transferable MDR plasmid among human pathogens poses a public health crisis and needs to be continuously monitored according to the One Health approach (https://www.cdc.gov/onehealth/basics/index.html).

## MATERIALS AND METHODS

### Bacterial isolation and antimicrobial susceptibility testing.

Carbapenem- and tigecycline-resistant Klebsiella aerogenes NUITM-VK5 was isolated from the Kim-Nguu River in Hanoi, Vietnam, in March 2021. Environmental water samples were collected and cultured using Luria-Bertani (LB) broth containing 4 mg/liter of meropenem at 37°C overnight and then further selected and isolated using CHROMagar Col-Apse (CHROMagar Microbiology) containing 4 mg/liter of tigecycline. Bacterial species identification was performed using the MALDI Biotyper (Bruker). Antimicrobial susceptibility testing (AST) using Escherichia coli ATCC 25922 as quality control was performed with agar dilution (other than colistin) and broth dilution methods (for colistin) according to the Clinical and Laboratory Standards Institute (CLSI) 2020 guidelines (21). The categorization as susceptible (S), intermediate (I), and resistant (R) was determined according to the MIC breakpoints. For tigecycline, AST was additionally performed in the presence or absence of 75 mg/liter of the efflux pump inhibitor 1-(1-naphthylmethyl)-piperazine (NMP) as used in the previous study (7).

### Whole-genome sequencing and subsequent bioinformatics analysis.

Whole-genome sequencing of K. aerogenes NUITM-VK5 was performed using MiSeq (Illumina) with MiSeq reagent kit v2 (300-cycle) and MinION (Oxford Nanopore Technologies) with the R9.4.1 flow cell. The library for Illumina sequencing (paired-end, insert size of 300 to 800 bp) was prepared using the Nextera XT DNA Library Prep kit, and the library for MinION sequencing was prepared using a rapid barcoding kit (SQK-RBK004). Illumina reads were assembled *de novo* using Shovill v1.1.0 (https://github.com/tseemann/shovill) with default parameters, resulting in the draft genome (accession no. BPFV01000000). MinION reads were basecalled using Guppy v4.2.2 with the high-accuracy mode and were assembled *de novo* using Canu v2.1.1 (https://github.com/marbl/canu) with default parameters. The overlap region in the assembled contig was detected using LAST (https://gitlab.com/mcfrith/last) and was trimmed manually. Sequencing errors were corrected by Racon v1.4.13 (https://github.com/isovic/racon) twice with default parameters using MinION reads and then corrected by Pilon v1.20.1 (https://github.com/broadinstitute/pilon/wiki) twice with default parameters using Illumina reads, resulting in a circular plasmid, pNUITM-VK5_mdr (accession no. LC633285).

Genome and plasmid sequences were annotated using the DFAST server (https://dfast.nig.ac.jp). Sequence type (ST) by multilocus sequence typing (MLST) analysis was determined according to the PubMLST protocol and database (https://pubmlst.org/organisms/klebsiella-aerogenes). Plasmid replicon type and antimicrobial resistance (AMR) genes were detected using PlasmidFinder v2.1 and ResFinder v4.1 with default parameters, respectively, on the CGE server (http://www.genomicepidemiology.org). Type IV secretion system (T4SS)-associated genes involved in conjugation were detected by TXSScan v1.0 (https://github.com/macsy-models/TXSS) with default parameters. Mobile gene elements (MGEs) were detected using BLAST with the ISfinder database updated in October 2020 (https://github.com/thanhleviet/ISfinder-sequences). The circular representation of the plasmid was visualized using the CGView server (http://cgview.ca). Linear comparison of sequence alignment was performed using BLAST and visualized by Easyfig v.2.2.2 (http://mjsull.github.io/Easyfig/).

### Bacterial conjugation assay.

A bacterial conjugation assay was performed as follows. LB broth cultures of the donor K. aerogenes NUITM-VK5 and the recipient azide-resistant E. coli J53 (ATCC BAA-2731, F^−^ *met pro* Azi^r^) were mixed in a 1:10 ratio, spotted onto MacConkey agar, and then incubated at 37°C overnight. Subsequently, the mixed cells, including transconjugants, were suspended in LB broth and then plated onto MacConkey agar containing 1 mg/liter of tigecycline and 100 mg/liter of sodium azide after 10-fold serial dilution and incubated at 37°C overnight. AMR genes, *bla*_NDM_, *tet*(X), and *tmexCD-toprJ*, of transconjugants were detected by colony PCR using the following primer sets: NDM_F, GGTTTGGCGATCTGGTTTTC; NDM_R, CGGAATGGCTCATCACGATC; tetX_F, CCCGAAAATCGWTTTGACAATCCTG; tetX_R, GTTTCTTCAACTTSCGTGTCGGTAAC; tmexC_F, TGGCGGGGATCGTGCTCAAGCGCAC; tmexC_R, CAGCGTGCCCTTGCKCTCGATATCG. The PCR amplification consisted of the initial denaturation for 3 min at 95°C, followed by 35 cycles of denaturation for 30 s at 95°C, annealing for 30 s at 56°C (for *bla*_NDM_) or 54°C [for *tet*(X) and *tmexCD-toprJ*], and extension for 30 s at 72°C, and then the final extension for 5 min at 72°C. Synthetic DNAs of *bla*_NDM-1_ (accession no. FN396876) and *tet*(X) (accession no. MK134376) and purified genomic DNA from *tmexCD1-toprJ1*-harboring Klebsiella pneumoniae strain MH15-269M (9) were used as positive controls. Purified water was used as a negative control.
