# Supplementary material for: A Transferable IncC-IncX3 Hybrid Plasmid Cocarrying blaNDM-4, tet(X), and tmexCD3-toprJ3 Confers Resistance to Carbapenem and Tigecycline
Source: mSphere. 2021 Aug 4;6(4):e00592-21. doi: 10.1128/mSphere.00592-21 (PMC8386453; doi:10.1128/mSphere.00592-21)
Supplement: FIG S1 [file msphere.00592-21-sf001.pdf]

Fig. S1

A
Tet(X)

```
BPFV01000000    MSNKEKQMNLLSDKNVAIIGGGPVGLTMAKLLQQNGIDVSVYERDNDREARIFGGTLDLH
MK134376        MSNKEKQMNLLSDKNVAIIGGGPVGLTMAKLLQQNGIDVSVYERDNDREARIFGGTLDLH
                ************************************************************

BPFV01000000    KGSGQEAMKKAGLLQTYYDLALPMGVNIADEKGNILSTKNVKPENRFDNPEINRNDLRAI
MK134376        KGSGQEAMKKAGLLQTYYDLALPMGVNIADEKGNILSTKNVKPENRFDNPEINRNDLRAI
                ************************************************************

BPFV01000000    LLNSLENDTVIWDRKLVMLEPGKKKWTLTFENKPSETADLVIIANGGMSKVRKFVTDTEV
MK134376        LLNSLENDTVIWDRKLVMLEPGKKKWTLTFENKPSETADLVIIANGGMSKVRKFVTDTEV
                ************************************************************

BPFV01000000    EETGTFNIQADIHHPEVNCPGFFQLCNGNRLMAAHQGNLLFANPNNNGALHFGISFKTPD
MK134376        EETGTFNIQADIHHPEVNCPGFFQLCNGNRLMAAHQGNLLFANPNNNGALHFGISFKTPD
                ************************************************************

BPFV01000000    EWKNQTQVDFQNRNSVVDFLLKEFSDWDERYKELIRVTSSFVGLATRIFPLGKSWKSKRP
MK134376        EWKNQTQVDFQNRNSVVDFLLKEFSDWDERYKELIRVTSSFVGLATRIFPLGKSWKSKRP
                ************************************************************

BPFV01000000    LPITMIGDAAHLMPPFAGQGVNSGLMDALILSDNLTNGKFNSIEEAIENYEQQMFAYGRE
MK134376        LPITMIGDAAHLMPPFAGQGVNSGLMDALILSDNLTNGKFNSIEEAIENYEQQMFIYGKE
                ******************************************************* **:*

BPFV01000000    AQAESIINETEMFSLDFSFQKLMNL
MK134376        AQEESTQNEIEMFKPDFTFQQLLNV
                ** **  ** ***. **:**:*:*:
```

B
TMexC3

```
BPFV01000000    MNKFREWITFSVISCLVAVTLVGCDKPEEQGEEAPAREVDVLSVQTEPFTVVAELPGRIE
CP066833        MNKFREWITFSVISCLVAVTLVGCDKPEEQGEEAPAREVDVLSVQTEPFTVVAELPGRIE
                ************************************************************

BPFV01000000    PVRVAEVRARVAGIVLKRTFEEGADVKAGDVLFQIDPAPFKAALSRAQGELARAEAQLFQ
CP066833        PVRVAEVRARVAGIVLKRTFEEGADVKAGDVLFQIDPAPFKAALSRAQGELARAEAQLFQ
                ************************************************************

BPFV01000000    AQAMVRRYEPLVKINAVSQQDFDNAKAALQSAQADKRSAQANVETARLDLGYAEVRAPIA
CP066833        AQAMVRRYEPLVKINAVSQQDFDNAKAALQSAQADKRSAQANVETARLDLGYAEVRAPIA
                ************************************************************

BPFV01000000    GRIGRAHVTEGALVGQGEATLLARIQQLDPVYADFTQPAADALRLRAAIAEGKVAGASDQ
CP066833        GRIGRAQVTEGALVGQGEATLLARIQQLDPVYADFTQPAADALRLRAAIAEGKVAGASDQ
                ******:*****************************************************

BPFV01000000    PLSLRVDGTDIESKGMLLFTDISVDRSTGQIALRGQFDNPEGVLLPGMYVRVRTPQGLNQ
CP066833        PLSLRVDGTDIESKGTLLFTDISVDRSTGQIALRGQFDNPEGVLLPGMYVRVRTPQGLNQ
                *************** ********************************************

BPFV01000000    NAILVPQRAVQRSADGQASVMLLGEGDTVEVRQVTTGAMQGSRWQISEGLQAGDKVITSS
CP066833        NAILVPQRAVQRSADGQASVMLLGEGDTVEVRQVTTGAMQGSRWQISEGLQAGDKVITSS
                ************************************************************

BPFV01000000    LAAIRPGAKVIPREQGAAEKAPQSQTQ
CP066833        LAAIRPGAKVIPREQGAAEKAPQSQAQ
                *************************:*
```

C
TMexD3

```
BPFV01000000    MPLFFIRRPNFAWVVALFISLGGLLVIPFLPVAQYPNVAPPQITVTATYPGASAQVLTDS
CP066833        MPLFFIRRPNFAWVVALFISLGGLLVIPFLPVAQYPNVAPPQITVTATYPGASAQVLTDS
                ************************************************************

BPFV01000000    VTSVIEEELNGAKNLLYFESTSNANGIAEITVTFQPGTDPELAQVDVQNRLKKAEARMPQ
CP066833        VTSVIEEELNGAKNLLYFESTSNANGIAEITVTFQPGTDPELAQVDVQNRLKKAEARMPQ
                ************************************************************

BPFV01000000    AVLTLGIQTEQATAGFLLIYALSYTDGDKDSDVTALADYAARSINNEIRRVPGVGKLQFF
CP066833        AVLTLGIQTEQATAGFLLIYALSYTDGDKDSDVTALADYAARSINNEIRRVPGVGKLQFF
                ************************************************************

BPFV01000000    ASEAAMRVWIDPQKLVGYGLSIDDVNNAIRAQNVQVPAGAFGSTPGSSEQELTATLAVKG
CP066833        ASEAAMRVWIDPQKLVGYGLSIDDVNNAIRAQNVQVPAGAFGSTPGSSEQELTATLAVKG
                ************************************************************

BPFV01000000    TLDNPQEFAAIVLRANQDGSRLTLGDVARIEVGSQDYNFGSRQDGKPAVAAAVQLSPGAN
CP066833        TLDNPQEFAAIVLRANQDGSRLTLGDVARIEVGSQDYNFGSRQDGKPAVAAAVQLSPGAN
                ************************************************************

BPFV01000000    AIQTAEAVKQRLTELSANFPDNVEFSVPYDTSRFVDVAIDKVIMTLIEAMVLVFLVMFLF
CP066833        AIQTAEAVKQRLTELSANFPDNVEFSVPYDTSRFVDVAIDKVIMTLIEAMVLVFLVMFLF
                ************************************************************

BPFV01000000    LQNVRYTLIPSIVVPVCLLGTLTFMYLLGFSVNMMTMFGMVLAIGILVDDAIVVVENVER
CP066833        LQNVRYTLIPSIVVPVCLLGTLTFMYLLGFSVNMMTMFGMVLAIGILVDDAIVVVENVER
                ************************************************************

BPFV01000000    IMAEEGLAPVPATIKAMGQVSGAIIGITLVLSAVFLPLAFMAGSVGVIYQQFSLSLAVSI
CP066833        IMAEEGLAPVPATIKAMGQVSGAIIGITLVLSAVFLPLAFMAGSVGVIYQQFSLSLAVSI
                ************************************************************

BPFV01000000    LFSGFLALTFTPALCATLLKPIPVGHHEKTGFFGWFNRKFTSLTSRYTKLNDKLVPRAGR
CP066833        LFSGFLALTFTPALCATLLKPIPVGHHEKTGFFGWFNRKFTSLTSRYTKLNDKLVPRAGR
                ************************************************************

BPFV01000000    VMFIYLGVVVLMGFLYMRLPESFVPVEDQGYMIVDIQLPPGATRERTSAAGGELESFLMA
CP066833        VMFIYLGVVVLMGFLYMRLPESFVPVEDQGYMIVDIQLPPGATRERTSAAGGELESFLMA
                ************************************************************

BPFV01000000    REAVQTTFLLFGFSFSGMGENAAIAFPLLKDWSERDSSQSPEAESVAVNEHFANLDDGAI
CP066833        REAVQTTFLVLGFSFSGMGENAAIAFPLLKDWSERDSSQSPEAESVAVNEHFANLDDGAI
                *********::*************************************************

BPFV01000000    MSVPPPPIEGLGNSGGFALRLQDRAGLGRDALLAARDEVLGKVNGNPKFLYAMMEGLAEA
CP066833        MSVPPPPIEGLGNSGGFALRLQDRAGLGRDALLAARDEVLGKVNGNPKFLYAMMEGLAEA
                ************************************************************

BPFV01000000    PQLRLVIDREQARTLGVSFEAISSALSTAFGSSVINDFANAGRQQRVVVQAEQAERMTPE
CP066833        PQLRLVIDREQARTLGVSFEAISSALSTAFGSSVINDFANAGRQQRVVVQAEQAERMTPE
                ************************************************************

BPFV01000000    SVLRLHVPNDSGSLVPLSAFVTTSWEEGPVQVARYNGYPSIRIAGDAAPGVSTGEAMLEL
CP066833        SVLRLHVPNDSGSLVPLSAFVTTSWEEGPVQVARYNGYPSIRIAGDAAPGVSTGEAMLEL
                ************************************************************

BPFV01000000    ERIAAELPEGIGYEWTGLSYQERVASGQATMLFALAITVVFLLLVALYESWAIPLTVMLI
CP066833        ERIAAELPEGIGYEWTGLSYQERVASGQATMLFALAITVVFLLLVALYESWAIPLTVMLI
                ************************************************************

BPFV01000000    VPVGALGAVLAVTAIGLPNDVYFKVGLITVIGLAAKNAILIVEFAKDLWEDGYSLRDAAV
CP066833        VPVGALGAVLAVTAIGLPNDVYFKVGLITVIGLAAKNAILIVEFAKDLWEDGYSLRDAAV
                ************************************************************

BPFV01000000    EAARLRFRPIIMTSMAFMLGVVPLAIATGAGAASQRALGTGVLGGMLSATMLGVIFVPIF
CP066833        EAARLRFRPIIMTSMAFMLGVVPLAIATGAGAASQRALGTGVLGGMLSATMLGVIFVPIF
                ************************************************************

BPFV01000000    FVWVLSLLRTKPQQTDNHPLHKAE
CP066833        FVWVLSLLRTKPQQTDNHPLHKAE
                ************************
```
